# Health inequalities for older people from minority ethnic groups receiving palliative care and end of life care: A scoping review protocol

**DOI:** 10.1371/journal.pone.0285109

**Published:** 2023-05-02

**Authors:** Narin Aker, Rachael Frost, Kate Walters, Emily West, Nathan Davies

**Affiliations:** 1 Research Department of Primary Care and Population Health, University College London, London, United Kingdom; 2 Division of Psychiatry, Marie Curie Palliative Care Research Department, University College London, London, United Kingdom; Xiamen University - Malaysia Campus: Xiamen University - Malaysia, MALAYSIA

## Abstract

**Objective:**

The proposed scoping review aims to explore international literature on how older people from minority ethnic groups engage with and utilise palliative and end of life care, investigate the barriers and facilitators, and compare how this varies between ethnicities and health conditions.

**Introduction:**

Minority ethnic groups make up substantial parts of the populations of many countries around the world. Research has found that there are disparities in access to palliative care and end of life care among minority ethnic groups. Language barriers, cultural values, and socio-demographic factors have been cited as preventing access to quality palliative and end of life care. However, it is unclear how these barriers and inequalities differ across different minority ethnic groups in different countries, and across different health conditions within these groups.

**Inclusion criteria:**

The population will be older people of different minority ethnic groups who are receiving palliative or end of life care, family caregivers, and health and social care professionals. The sources of information will include quantitative, qualitative and mixed methods research, and sources that focus on minority ethnic groups’ interactions with palliative and end of life care.

**Methods:**

A scoping review guided by the Joanna Briggs Institute Manual for Evidence Synthesis. Searches of MEDLINE, Embase, PsycInfo, CINAHL, Scopus, Web of Science, Assia, and the Cochrane Library will be conducted. Citation tracking, reference list checking and grey literature searches will be undertaken. Data will be extracted, charted and summarised descriptively.

**Implications:**

This review will highlight the health inequalities present in palliative and end of life care, the research gaps in understudied minority ethnic populations, locations where further study is required, and how barriers and facilitators differ across different ethnic groups and health conditions. The results of this review will be shared with stakeholders and will provide evidence-based recommendations for inclusive palliative and end of life care.

## Introduction

Palliative care is an approach to care that improves the quality of life of individuals and their families who are facing problems related to serious and life-threatening illnesses. It aims to prevent and relieve suffering through the identification, assessment and treatment of pain and other problems, including the physical, psychosocial and spiritual [1]. Palliative care is delivered by a range of health and social care professionals in hospitals, hospices and the community, who may be working within a specialist palliative care team [2], or providing generalist palliative care as part of standard clinical practice [3]. End of life care is the palliative care a person receives when they are close to the end of their life [4]. In the UK, end of life is often considered as the final 12 months of life [5]. However, for some conditions such as dementia, it may not be possible to provide an accurate prognosis, and end of life can be seen as an extended period of time [6].

A minority ethnic group is a particular ethnic group (a group of people with a shared culture, tradition, language, history, etc.) living in a country where most people are from a different ethnic group [7]. Many people from minority ethnic groups experience health inequalities. These are unfair and preventable differences in the health status between different groups of people [8], which can be caused by the unequal distribution of health determinants, including people’s physical environments, social support networks, income or social status, and access to health services [9].

Research has found that there are differences in accessing palliative care among minority ethnic groups [10]. Barriers that contribute to low engagement with advance care planning in people from minority ethnic groups include socio-demographic factors; health status, health literacy and experiences with health care systems; cultural values; and spirituality or religion [11]. Language barriers can also be a barrier for older people accessing palliative care, which can impact use of services and result in adverse experiences [12], making it difficult to establish trust with care professionals [13]. In many minority ethnic communities, people generally care for family members for longer than the general population does [14], and there may be a sense of responsibility or moral duty for looking after older family members at home [15]. Despite this, family caregivers from minority ethnic groups are less likely to access care services for the family member they are caring for than White family caregivers [16].

The barriers faced by older people from minority ethnic groups and family caregivers have resulted in a disparity in access to healthcare. “Access” to healthcare is the ability to receive healthcare services, including prevention, diagnosis, treatment, and management [17] for any health condition or concerns. Expanding on this, the extent of “access” also depends on financial, organisational and social or cultural barriers that may limit people’s use of services. Therefore, access should be measured in terms of the affordability, physical accessibility and acceptability of services in the context of the different groups in society and not just their existence [18].

### Rationale

The aforementioned evidence shows that health inequalities exist towards the end of life for many older people from minority ethnic groups, as well as barriers to accessing palliative care. However, at present it is unclear how these barriers and inequalities differ across different minority ethnic groups in different countries, and across different health conditions within these groups. It is also unclear how the identified barriers and inequalities have been explored in research and literature. A scoping review approach will be used to identify the available evidence relating to these populations, who are often underrepresented in research [19], highlight what the gaps in the evidence are, and identify areas for future research.

A search for existing and in-progress reviews on this topic was conducted in April 2022 and there were none identified. A formal review of reviews was not undertaken.

### Review question

How do older people from minority ethnic groups experience palliative and end of life care, and how does this vary between different ethnicities, countries, and health conditions?

#### Objectives

The overarching objectives of this proposed scoping review are:

1. Identify how older adults of different minority ethnic groups engage with and utilise specialist or generalist palliative care and end of life care
2. Scope the landscape and nature of the existing evidence base available on palliative and end of life care for different ethnic groups and health conditions
3. Understand the inequalities, barriers and facilitators in accessing palliative care and end of life care experienced from the perspectives of older people, family caregivers and healthcare professionals
4. Compare how access and utilisation of palliative and end of life care varies between ethnicity and health conditions

## Methods

This protocol has been developed in line with the Joanna Briggs Institute (JBI) Manual for Evidence Synthesis [20] chapter 11.2: Development of a scoping review protocol. This manual was used to inform the structure, layout and content of this protocol. The PRISMA (Preferred Reporting Items for Systematic Reviews and Meta-Analyses) 2020 Checklist for systematic review protocols [21] was also used to help facilitate the development of this review protocol.

### Eligibility criteria

#### Participants

The population for the review will be:

- Older people of different minority ethnic groups who are receiving palliative care, end of life care, or care towards the end of life.
- Family/friend caregivers of older people from minority ethnic groups who are receiving palliative care, end of life care, or care towards the end of life.
- Health and social care professionals involved in palliative care for older people.

Papers that have a majority population (over 50%) of older people will be included. There is no universally accepted age that is considered ‘old’ among or within societies, but in many countries, 60 to 65 years is the age range generally used to define old age for statistical and administrative purposes. For the purpose of the review, ‘older people’ is defined as individuals aged over the age of 60.

A caregiver is an individual who regularly takes care of someone due to their illness, frailty or disability [22]. The review will focus on unpaid, adult family caregivers.

The specific ethnic groups included in the review will differ based on the country the research or resource is based in. Since different countries may refer to minority ethnic groups in different ways, the review will take into account self-defined minority status, and also note terms used in sources from different countries and include these in searches.

#### Concept

Sources focusing on minority ethnic groups’ interactions with palliative care (both specialist and generalist), end of life care, and care towards the end of life will be included. These interactions include which services are used, when they are used, who they are generally used by, and what enables people to access services or prevents people from accessing services.

Sources that explore the health inequalities faced by people from minority ethnic groups towards the end of life, including what inequalities are present and why they may occur, will be included. The outcome of the literature will identify patterns of engagement, barriers and facilitators to accessing palliative care, and what the health inequalities are for different minority ethnic groups.

#### Context

Literature included will not be limited by factors such as geographic location or healthcare setting. This broad approach will allow for more comparisons between the different minority ethnic groups studied in the available literature and the different contexts, and will more clearly highlight under-researched areas across different settings.

#### Types of sources

The sources of information will remain open to allow for the inclusion of all types of evidence, including grey literature (documents produced outside of traditional means of publishing and distribution). In addition to providing a more comprehensive evidence base, the inclusion of grey literature will help avoid positive results publication bias, and will provide another way of identifying relevant published research cited within that may be missing from the included databases [23]. Examples of sources to be included in this review are primary research studies, systematic reviews, guidelines, audits and reports, theses and dissertations, preprints, and others. Qualitative, quantitative and mixed methods sources will be included.

### Exclusion criteria

- Paid caregivers and young caregivers under the age of 18 will be excluded because they have different responsibilities and experiences to those that adult family caregivers may have.
- People receiving palliative care, end of life care, or care towards the end of life who are under the age of 60 will be excluded. Studies including both younger and older adults, or minority ethnic and majority ethnic participants, will only be included if the data from the relevant subgroup is presented or can be extracted separately.
- Conference abstracts, study/review protocols, literature reviews with no findings/results, and opinion pieces will be excluded. However, conference abstracts and study/review protocols will be included if they are relevant to the research question and related published work can be found.
- Sources that do not focus on older people (with less than 50% of study population) will be excluded.
- Sources that do not present results that focus on palliative or end of life care will be excluded.

### Search strategy

A three-step search strategy will be used, as recommended by the JBI Manual for Evidence Synthesis [20]. A preliminary search of three online databases (MEDLINE, Embase, and Web of Science) will be undertaken using the following concepts: minority ethnic groups, older adults, palliative care, and health inequalities. Search terms will be refined based on search output and with input from all authors and a research librarian. Following preliminary searches, a full list of search terms and search strategy will be developed (please see S1 Table for an example full search strategy). Searches using all identified keywords and index terms will then be undertaken across all the databases to be included. In addition to the above three databases CINAHL, PsycInfo, Assia, Scopus, and the Cochrane Library will be used. The reference lists of all the papers to be included in the review will be searched for additional sources. Citation tracking will be undertaken using Google Scholar. Authors of publications may be contacted for further information if necessary.

A search for grey literature will be undertaken. This will be guided by the Grey Matters tool for searching health-related grey literature [24], and further informed by the Index of Grey Literature and Alternative Sources and Resources [25]. In addition, the Social Care Online database, British library EThOS (e-theses online service), and the websites of national and international palliative care charities will be searched. Supplemental key word searches on Google will be conducted. Patient and Public Involvement (PPI) members will also be asked for suggestions as to other sources of grey literature they may be aware of. Authors of grey literature may be contacted for further information if necessary.

There will be no date limit to the searches, as there is no date for cut-off that is meaningful across many countries. Non-English resources will not be excluded from the searches, and it will be endeavoured to translate relevant non-English papers where resources permit, by working with colleagues across the team and department who speak various languages.

While search terms will be identified prior to beginning the review, the JBI Manual highlights that the search for a scoping review may be iterative as reviewers become more familiar with the evidence base.

### Evidence selection

Evidence selection for the review is to be based on the inclusion criteria specified in this protocol. Search results will be deduplicated using EndNote X9 [26] and exported into Rayyan [27] for screening. Evidence selection for published research, both at the title/abstract screening and full-text screening stages, will be performed by the first reviewer (NA), with a second reviewer (PN) reviewing a random 10% of title/abstracts and full texts. The titles and abstracts of papers will be read during the title/abstract screening stage, and whole articles will be read during the full text screening stage. All reviewers have been trained in scoping reviews and the review is supervised by two senior and experienced reviewers (ND and RF). The reviewers will meet during the review process to discuss challenges and uncertainties related to study selection, and to refine the inclusion criteria or search strategy if necessary. Disagreements between reviewers will be solved through discussion or if necessary, by the input of a third reviewer. The selection process and reasons for exclusion will be presented in a PRISMA 2020 flow diagram for new systematic reviews [28] and will be narratively summarised.

Any necessary modifications to the eligibility criteria or definitions will be made to ensure that they are robust enough to capture the literature needed.

### Data extraction

The review data will be charted using a data extraction tool developed by the first reviewer (NA). Some sections may not be applicable to every source included. In this case, the relevant sections will be completed. The key information that will be charted includes:

- Author(s)
- Year of publication
- Country of origin
- Journal or source details
- Aims/purpose
- Qualitative or quantitative study
- Study population
- Sample size
- Participant age groups, ethnic groups, and health conditions
- Intervention details (if applicable)
- Study setting
- Recruitment
- Methods
- Methods of analysis
- Key findings
- Conclusions
- Strengths and limitations

The extraction tool will be trialled on two sources to ensure all relevant information is able to be extracted, and this will be checked by the second reviewer. In order to map the existing evidence, quality assessment of included sources will not be undertaken, in line with scoping review methods [29].

### Data analysis and presentation

Key information from each study will be presented using a table, with overall characteristics of the literature narratively summarised. Data for different minority ethnic groups will be compared to each other. The context of minority ethnic groups within different countries will summarised narratively and discussed. Gaps in the literature will also be identified by exploring the topics on palliative care for older people from minority ethnic groups that have been studied. We will highlight the conclusions from the literature, including suggestions for further research.

The PRISMA Extension for Scoping Reviews (PRISMA-ScR) [30] checklist will be used to support the write-up and presentation of the findings, and data extracted from the sources of evidence will be presented as a table. This will be supported by charts and graphs highlighting key comparisons and frequency counts that relate to the review question and objective, such as age, ethnic background, country of source origin, health conditions, type of evidence and healthcare settings. Descriptive summaries will accompany all tables and charts, and will describe how the results relate to the review objective and question. Areas where there are substantial gaps in the literature will be highlighted.

## Implications

### Stakeholder engagement

Initial findings will be presented to PPI members and stakeholders, such as palliative care charities and patient and carer organisations, in the form of lay summaries, diagrams, and infographics. Feedback from PPI members will be used to further inform the synthesis, highlight gaps in the literature, provide recommendations for future research and explore avenues for wider dissemination of findings.

### Impact

This scoping review will increase the understanding of the palliative and end of life care experiences of people from minority ethnic backgrounds and family caregivers. This will highlight the health inequalities present in palliative care for minority ethnic groups and how this differs between groups, with an in-depth exploration of the impact of context of country and health condition. It will also provide an insight into how other countries address health inequalities and provide palliative and end of life care. This will provide evidence-based recommendations as to how these services can be more inclusive and where the gaps currently lie in providing high quality palliative and end of life care for these populations.

## Supporting information

S1 Table Search strategy. This is the initial search strategy used on MEDLINE database.
(DOCX)

S1 Checklist PRISMA-P checklist. A PRISMA checklist for systematic review protocols.
(DOCX)
