# Supplementary material for: Health inequalities for older people from minority ethnic groups receiving palliative care and end of life care: A scoping review protocol
Source: PLoS One. 2023 May 2;18(5):e0285109. doi: 10.1371/journal.pone.0285109 (PMC10153691; doi:10.1371/journal.pone.0285109)
Supplement: S1 Table — This is the initial search strategy used on MEDLINE database. (DOCX) [file pone.0285109.s001.docx]

**S1 Table: Search strategy**

Ovid MEDLINE(R) ALL <1946 to April 08, 2022>

| 1 | "Ethnic and Racial Minorities"/ | 282 |
| --- | --- | --- |
| 2 | "ethnic minorit*".ab,kw,ti. | 13720 |
| 3 | "minority ethnic".ab,kw,ti. | 1667 |
| 4 | minority racial.ab,kw,ti. | 226 |
| 5 | racial.ab,kw,ti. | 50191 |
| 6 | "racial minorit*".ab,kw,ti. | 1606 |
| 7 | "minorit*".ab,kw,ti. | 81702 |
| 8 | bme.ab,kw,ti. | 2871 |
| 9 | bame.ab,kw,ti. | 308 |
| 10 | "black*".ab,kw,ti. | 172157 |
| 11 | "british black*".ab,kw,ti. | 37 |
| 12 | black british.ab,kw,ti. | 100 |
| 13 | "asian*".ab,kw,ti. | 101344 |
| 14 | "british asian*".ab,kw,ti. | 103 |
| 15 | "asian british".ab,kw,ti. | 61 |
| 16 | "black american*".ab,kw,ti. | 1828 |
| 17 | "african american*".ab,kw,ti. | 59887 |
| 18 | "asian american*".ab,kw,ti. | 4561 |
| 19 | "latin*".ab,kw,ti. | 55168 |
| 20 | "hispanic*".ab,kw,ti. | 54852 |
| 21 | "pacific island*".ab,kw,ti. | 7017 |
| 22 | "middle east*".ab,kw,ti. | 17737 |
| 23 | "south america*".ab,kw,ti. | 27338 |
| 24 | "central america*".ab,kw,ti. | 6632 |
| 25 | "indigenous people*".ab,kw,ti. | 4230 |
| 26 | indigenous.ab,kw,ti. | 37259 |
| 27 | "ethnic*".ab,kw,ti. | 165996 |
| 28 | exp Racial Groups/ | 239973 |
| 29 | exp Ethnicity/ | 100682 |
| 30 | "ethnic group*".ab,kw,ti. | 39112 |
| 31 | "foreign*".ab,kw,ti. | 90141 |
| 32 | cultural diversity/ | 12509 |
| 33 | cross-cultural comparison/ | 27020 |
| 34 | "cross-cultur*".ab,kw,ti. | 16040 |
| 35 | exp Population Groups/ | 314428 |
| 36 | exp "health disparity, minority and vulnerable populations"/ | 104057 |
| 37 | "Transients and Migrants"/ | 13287 |
| 38 | "transcultur*".ab,kw,ti. | 2846 |
| 39 | exp "Emigrants and Immigrants"/ | 14627 |
| 40 | "migrant*".ab,kw,ti. | 22818 |
| 41 | "immigrant*".ab,kw,ti. | 28158 |
| 42 | Refugees/ | 12032 |
| 43 | "refugee*".ab,kw,ti. | 12947 |
| 44 | "asylum seeker*".ab,kw,ti. | 2086 |
| 45 | "seek* asylum".ab,kw,ti. | 258 |
| 46 | displaced people.ab,kw,ti. | 309 |
| 47 | Health Services, Indigenous/ | 3783 |
| 48 | Minority Groups/ | 16279 |
| 49 | "old* adult*".ab,kw,ti. | 105783 |
| 50 | "old* people*".ab,kw,ti. | 39860 |
| 51 | "old* person*".ab,kw,ti. | 14137 |
| 52 | exp Dementia/ | 189251 |
| 53 | Health Services for the Aged/ | 18137 |
| 54 | "elder*".ab,kw,ti. | 288745 |
| 55 | seniors.ab,kw,ti. | 8367 |
| 56 | "senior citizen*".ab,kw,ti. | 1639 |
| 57 | Geriatrics/ | 30995 |
| 58 | Geriatric Nursing/ | 13796 |
| 59 | Frailty/ | 6317 |
| 60 | "frail*".ab,kw,ti. | 29958 |
| 61 | Palliative Care/ | 59917 |
| 62 | "palliative treatment*".ab,kw,ti. | 7813 |
| 63 | palliative.ab,kw,ti. | 70512 |
| 64 | Palliative Medicine/ | 466 |
| 65 | palliative medicine.ab,kw,ti. | 2364 |
| 66 | "Hospice and Palliative Care Nursing"/ | 1741 |
| 67 | Hospices/ | 5456 |
| 68 | "hospice*".ab,kw,ti. | 14298 |
| 69 | "hospice care".ab,kw,ti. | 3753 |
| 70 | Death/ | 18937 |
| 71 | death.ab,kw,ti. | 790118 |
| 72 | dying.ab,kw,ti. | 37845 |
| 73 | exp Terminal Care/ | 55083 |
| 74 | terminal care.ab,kw,ti. | 2529 |
| 75 | end of life.ab,kw,ti. | 27097 |
| 76 | end of life care.ab,kw,ti. | 12645 |
| 77 | "end of life treatment*".ab,kw,ti. | 423 |
| 78 | Terminally Ill/ | 6747 |
| 79 | terminally ill.ab,kw,ti. | 5680 |
| 80 | "terminal illness*".ab,kw,ti. | 2270 |
| 81 | life-limiting.ab,kw,ti. | 2485 |
| 82 | exp Advance Care Planning/ | 10632 |
| 83 | exp Advance Directives/ | 7571 |
| 84 | exp health inequities/ | 18985 |
| 85 | Culturally Competent Care/ | 2022 |
| 86 | "health* inequalit*".ab,kw,ti. | 7534 |
| 87 | "health* equalit*".ab,kw,ti. | 145 |
| 88 | "health* equit*".ab,kw,ti. | 6087 |
| 89 | "health* inequit*".ab,kw,ti. | 3574 |
| 90 | Health Equity/ | 2632 |
| 91 | Healthcare Disparities/ | 20972 |
| 92 | Health Services Accessibility/ | 82930 |
| 93 | "utili?*".ab,kw,ti. | 971327 |
| 94 | "access*".ab,kw,ti. | 608421 |
| 95 | chinese.ab,kw,ti. | 287522 |
| 96 | "india*".ab,kw,ti. | 187309 |
| 97 | "pakistan*".ab,kw,ti. | 26961 |
| 98 | "bangladesh*".ab,kw,ti. | 18430 |
| 99 | "african*".ab,kw,ti. | 164500 |
| 100 | "caribbean*".ab,kw,ti. | 17221 |
| 101 | "arab*".ab,kw,ti. | 151568 |
| 102 | "traveller*".ab,kw,ti. | 5570 |
| 103 | "roma*".ab,kw,ti. | 25193 |
| 104 | "gyps*".ab,kw,ti. | 6975 |
| 105 | "jew*".ab,kw,ti. | 14282 |
| 106 | "muslim*".ab,kw,ti. | 5956 |
| 107 | "sikh*".ab,kw,ti. | 428 |
| 108 | "buddhis*".ab,kw,ti. | 1564 |
| 109 | "hindu*".ab,kw,ti. | 2166 |
| 110 | 1 or 2 or 3 or 4 or 5 or 6 or 7 or 8 or 9 or 10 or 11 or 12 or 13 or 14 or 15 or 16 or 17 or 18 or 19 or 20 or 21 or 22 or 23 or 24 or 25 or 26 or 27 or 28 or 29 or 30 or 31 or 32 or 33 or 34 or 35 or 36 or 37 or 38 or 39 or 40 or 41 or 42 or 43 or 44 or 45 or 46 or 47 or 48 or 95 or 96 or 97 or 98 or 99 or 100 or 101 or 102 or 103 or 104 or 105 or 106 or 107 or 108 or 109 | 1648717 |
| 111 | 49 or 50 or 51 or 52 or 53 or 54 or 55 or 56 or 57 or 58 or 59 or 60 | 632557 |
| 112 | 61 or 62 or 63 or 64 or 65 or 66 or 67 or 68 or 69 or 70 or 71 or 72 or 73 or 74 or 75 or 76 or 77 or 78 or 79 or 80 or 81 or 82 or 83 | 946010 |
| 113 | 84 or 85 or 86 or 87 or 88 or 89 or 90 or 91 or 92 or 93 or 94 | 1606107 |
| 114 | 110 and 111 and 112 and 113 | 464 |
